# Maternal effect determines drought resistance of eggs in the predatory mite *Phytoseiulus persimilis*

**DOI:** 10.1007/s00442-019-04556-0

**Published:** 2019-11-26

**Authors:** Sophie Le Hesran, Thomas Groot, Markus Knapp, Tibor Bukovinszky, Jovano Erris Nugroho, Giuditta Beretta, Marcel Dicke

**Affiliations:** 1 Koppert BV, Veilingweg 14, Postbus 155, 2650 AD Berkel en Rodenrijs, The Netherlands; 2 Laboratory of Entomology, Wageningen University, PO Box 16, 6700 AA Wageningen, The Netherlands

**Keywords:** Relative humidity, Egg hatching, Oviposition, Survival, Phytoseiidae

## Abstract

The ability of an organism to adapt to short-term environmental changes within its lifetime is of fundamental importance. This adaptation may occur through phenotypic plasticity. Insects and mites, in particular, are sensitive to changes in temperature and humidity, especially during the juvenile stages. We studied the role of phenotypic plasticity in the adaptation of eggs to different relative humidity conditions, in the predatory mite *Phytoseiulus persimilis*, used worldwide as a biological control agent of the spider mite *Tetranychus urticae*. The biocontrol efficacy of *P. persimilis* decreases under dry conditions, partly because *P. persimilis* eggs are sensitive to drought. We exposed *P. persimilis* adult females from two different strains to constant and variable humidity regimes and evaluated the hatching rate of their eggs in dry conditions, as well as the survival and oviposition rates of these females. Whereas the eggs laid by *P. persimilis* females exposed to constant high humidity did not survive in dry conditions, females exposed to constant low humidity started laying drought-resistant eggs after 24 h of exposure. Survival and oviposition rates of the females were affected by humidity: females laid fewer eggs under constant low humidity and had a shorter lifespan under constant high and constant low humidity. The humidity regimes tested had similar effects across the two *P. persimilis* strains. Our results demonstrate that transgenerational phenotypic plasticity, called maternal effect, allows *P. persimilis* females to prepare their offspring for dry conditions.

**Electronic supplementary material:**

The online version of this article (10.1007/s00442-019-04556-0) contains supplementary material, which is available to authorized users.

## Introduction

Long-term adaptations, through natural selection, may lead to a match between organisms and their environment (Darwin 1859). Short-term variation in environmental factors, however, can disrupt this match and negatively affect the survival and fitness of an organism (Nussey et al. 2007; Whitman and Agrawal 2009). Understanding how organisms adapt to short-term environmental changes and the consequences of this adaptation at the population level is necessary to predict population dynamics in stressful environments.

Terrestrial insects and mites, in particular, are highly sensitive to changes in temperature and humidity, because they are poikilothermic organisms (Gotoh et al. 2014) with small body size and a large surface-area-to-volume ratio (Gibbs 2002; Gefen et al. 2006). Under variable abiotic conditions, they face two main physiological challenges: avoiding harmful body temperatures, and retaining sufficient water while maintaining gas exchange (Potter and Woods 2012). One solution to these challenges is phenotypic plasticity, defined as the ability of an individual to display a range of different phenotypes in multiple environments (DeWitt et al. 1998). Phenotypic plasticity allows individuals to adjust to environmental changes in real time (Whitman and Agrawal 2009) and includes morphological, behavioural, physiological, and molecular adaptations (Price et al. 2003). These plastic adaptations can vary significantly depending on many factors, like the developmental stages of insects and mites (Whitman and Agrawal 2009; Potter and Woods 2012; Ghazy et al. 2016; Fischer and Kirste 2018; Mutamiswa et al. 2019). Early developmental stages such as eggs and larvae are often considered more vulnerable to environmental stresses, because of their limited dispersal ability and their small size (Schausberger 1998; Montserrat et al. 2007; Walzer et al. 2007; Ferrero et al. 2010; Potter and Woods 2012; Döker et al. 2016; Torres-Campos et al. 2016). Although many studies on insects and mites have focused on the sensitivity of the egg stage to extreme temperature and humidity conditions (Colloff 1987; Sota and Mogi 1992; Schausberger 1998; Williams et al. 2004; Yoder et al. 2004; Walzer et al. 2007; Ferrero et al. 2010; Potter and Woods 2012; Le Hesran et al. 2019), most of these studies have only exposed the eggs to stressful abiotic conditions, while the females that produced them were kept under favourable conditions. In various species, it has been shown that a mother can change the type of eggs that she lays or can program a developmental switch in her offspring, so that it may better endure adverse environmental conditions (Saunders 1966; Margolies and Wrensch 1996; Mousseau and Dingle 1991; Fox et al. 1999; Fischer et al. 2003; Rahman et al. 2004; Yoder et al. 2006; Montserrat et al. 2007; Ross et al. 2011). For example, when females of the parasitic wasp *Nasonia vitripennis* (Walker) are exposed to short day length and low temperature, the majority of their larval offspring will enter diapause (Saunders 1966). This special case of transgenerational phenotypic plasticity is called maternal effect. It is defined as the causal influence of the maternal genotype or phenotype on the offspring’s phenotype (Wolf and Wade 2009). A maternal effect can also be considered as a ‘shared phenotype’ that affects both maternal and offspring fitness simultaneously (Rossiter 1991; Marshall and Uller 2007; Walzer and Schausberger 2015). How females find the most ‘adaptive’ strategy to ensure the survival of their offspring as well as their own survival in stressful conditions is, therefore, an essential question when studying maternal effects.

Moreover, these maternal adjustments of offspring phenotype may vary within and between populations, due to genetic variation for plasticity (Pigliucci 2005). For example, populations that experience the greatest extent of variability in humidity conditions are expected to be more plastic in traits that mitigate humidity stress (Valladares et al. 2014). Finally, although maternal effects are increasingly recognized for their role in adaptation to variable environments (Lorenzon et al. 2001; Marshall and Uller 2007; Van Asch et al. 2010), little is known about their impact on insects and mites under extreme temperature and humidity conditions.

In the present study, we focus on the effects of low relative humidity (RH) on the phytoseiid predatory mite *Phytoseiulus persimilis* Athias-Henriot (Acari: Phytoseiidae). *P. persimilis* is the most frequently applied predator for biological control of two-spotted spider mites (*Tetranychus urticae* Koch; Acari: Tetranychidae), and its efficacy is dependent on temperature and humidity conditions (Weintraub and Palevsky 2008). This predatory mite goes through five developmental stages: egg, larva, protonymph, deutonymph, and adult (Sabelis 1981). The egg stage is expected to be the most drought-sensitive life stage, because eggs cannot move, feed, or drink to compensate for water deficit. Eggs of *P. persimilis* do not survive at constant low humidity. This sensitivity of the egg stage is considered to be partly responsible for the low efficacy of *P. persimilis* as a biocontrol agent in dry conditions (Sabelis 1985; Croft et al. 1993; Schausberger 1998; Walzer et al. 2007; Ferrero et al. 2010; Döker et al. 2016). However, the effects of drought on other life stages of this predator are still unclear. To our knowledge, no study has focused on the impact of drought stress on adult females in *P. persimilis* or other phytoseiid mite species, and more specifically on the effects of this drought stress on the drought sensitivity of their eggs.

The three main objectives of this study were to: (1) investigate whether maternal strategies enhance the survival of *P. persimilis* eggs under stressful humidity conditions and estimate the plasticity of these strategies, (2) evaluate the effects of different humidity levels on *P. persimilis* adult females and (3) evaluate the degree of genetic variation for potential maternal strategies in *P. persimilis*.

We showed in a previous study that *P. persimilis* eggs have a different sensitivity to constant and variable humidity conditions (Le Hesran et al. 2019). We, therefore, exposed *P. persimilis* females to constant low, constant high, and variable humidity conditions. To investigate the potential maternal strategies promoting egg survival under dry conditions, we assessed, under low humidity, the hatching rate of the eggs laid by these females. To estimate the plasticity of these maternal strategies, we exposed the females to a sudden change in humidity conditions (see humidity treatment “variable 2”). To evaluate the effects of different humidity levels on *P. persimilis* females, we focused on their oviposition and survival rates, two traits determining population growth and biocontrol efficacy. As there may be trade-offs between fecundity and survivorship (Biro and Stamps 2008), it is important to study them simultaneously. Although *P. persimilis* females can live for more than 60 days (Amano and Chant 1977), we studied their survival rate over a period of 20 days (from 7 days old to 27 days old). Egg production of *P. persimilis* females at 25 °C starts within 24 h after mating and will continue for a period of 15–20 days (Schulten et al. 1978). Therefore, we considered these first 20 days as the most important part of a *P. persimilis* female’s life. We studied their oviposition rate over periods of 4 and 10 days. Finally, to evaluate the degree of genetic variation for potential maternal strategies, we compared the egg hatching, oviposition and survival rates of two different strains of *P. persimilis*, with different geographical origins and reared under different humidity conditions.

## Materials and methods

### Predatory mites

Two *P. persimilis* strains were used: a “commercial strain”, and a “mixed strain”. The commercial strain was obtained from a commercial mass rearing (Koppert Biological Systems), and reared in two Petri dishes, containing around 100 individuals each (see Le Hesran et al. 2019). The mixed strain was created by mixing individuals from five different strains (50 individuals per strain). Four of these strains were field collected in France, Italy, Israel, and Turkey (see Le Hesran et al. 2019). The fifth one was obtained from the same commercial mass rearing as the commercial strain (Koppert Biological Systems). In November 2016, 1 month after mixing these five strains, 150 individuals were randomly collected from this mix and transferred to a pot containing four faba bean plants (*Vicia faba* L.) infested with *T. urticae*. The pot was kept in a climate chamber (Panasonic Versatile Environmental Test Chamber MLR-352) at 55 ± 2% RH during the day (16 h) and 65 ± 2% RH during the night (8 h), at 25 °C (photoperiod L16:D8). The bean plants were replaced once a week and spider mites were provided as food twice a week. The pot with bean plants was placed in a tray filled with sunflower oil to prevent mite dispersal. The mixed strain was reared in these conditions during 17 months before the experiments started. To control for the influence of *P. persimilis* female age, we used even-aged cohorts of young adult females (7 days since the egg stage) for all experiments. These females were collected as eggs from the two strains, and kept for 7 days in two separate Petri dishes, in a climate cell at 70 ± 2% RH and 25 ± 1 °C. Inside the Petri dishes, an agar layer (agar powder, VWR Chemicals, 1/100 diluted) and a cucumber leaf disk infested with spider mites provided optimal conditions for the development of these eggs to adults.

### Humidity treatments

**Fig. 1:**
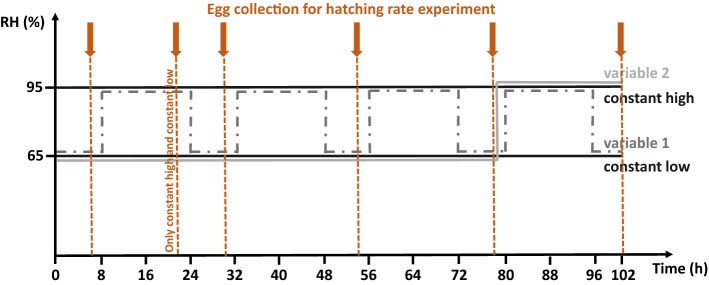
Four humidity treatments tested in this study. The arrows represent the time points when eggs were collected for the hatching rate experiment

We tested four humidity treatments: “constant low” (65% RH), “constant high” (95% RH), “variable 1” (successive cycles of 8 h at 65% RH and 16 h at 95% RH), and “variable 2” (78 h at 65% RH followed by 24 h at 95% RH) (Fig. 1). We showed in a previous study that only 39% of *P. persimilis* eggs survive at 65% RH and 25 °C (Le Hesran et al. 2019). We, therefore, considered that 65% RH was stressful enough for *P. persimilis* eggs and that these humidity conditions were likely to trigger a maternal effect in *P. persimilis* females.

One replicate consisted of 15–20 adult females per treatment and per strain. The females were placed in individual plastic cups (ø 3.5 cm, 2.8 cm deep), covered by a lid with a fine gauze (gauze-width 90 µm). To provide females with an oviposition substrate, a thin layer of cardboard with hairy surface facing up was fixed with a double-sided tape (Tesa^®^) at the bottom of each cup. For humidity treatment “constant low”, the cups were placed upside down on top of a wire platform (2.5 × 2.5 cm spacing) inside a closed plastic box (40 L × 25.5 W × 16.5 H cm). The relative humidity inside the box was regulated with a humidifier (Cigar Oasis Excel), to achieve constant 65% RH (average 64.7 ± 1.6% RH). For humidity treatment “constant high”, the cups were placed upside down on an agar layer at the bottom of a closed plastic box, in which the relative humidity was maintained at 95% RH (average 96.2 ± 1.7% RH). For humidity treatment “variable 1”, two additional plastic boxes were set up using the same methods: one box at 65% RH and one box at 95% RH. The cups were kept 8 h per day in the box at 65% RH (photoperiod L8:D0), and 16 h per day in the box at 95% RH (photoperiod L8:D8). For humidity treatment “variable 2”, the cups were kept during 78 h in the same box as humidity treatment “constant low”, followed by 24 h in the same box as humidity treatment “constant high”. All boxes containing the cups were placed in a climate cell at 70 ± 2% RH and 25 ± 1 °C (L16:D8 photoperiod). The females were provided with fresh *T. urticae* (larvae, nymphs and adults) ad libitum every day.

### Egg hatching

We collected freshly laid *P. persimilis* eggs (between 0 and 6 h old) after 6, 21, 30, 54, 78, and 102 h of female exposure to each humidity treatment (for humidity treatments “variable 1” and “variable 2”, we did not collect eggs after 21 h of female exposure). To achieve this, all eggs present in each cup were removed 6 h before collecting the eggs. 6 h later, the freshly laid eggs were collected from each cup and placed with a brush in a hole (ø 0.7 cm, 0.4 cm deep) in a platform made of polymethyl methacrylate (PMMA) (17.5 L × 15 W × 4.5 H cm) containing 30 holes (one egg per hole). Each hole had a fine gauze at the bottom (gauze-width 90 µm), to ensure contact with ambient air, and was then covered with a coverslip to prevent the larvae from escaping after hatching. The PMMA platforms (one platform per collection time, per strain, per humidity treatment) were placed in a climate chamber at 60 ± 1.6% RH, 25 ± 1.7 °C and L16:D8 photoperiod. Egg hatching rate (number of hatched eggs divided by total number of eggs in the platform) was recorded 72 h after placing the platforms in the climate chamber. A data logger (LogTag TRIX 8) was placed in the climate chamber and in each box containing the cups, to measure relative humidity and temperature. For each humidity treatment and each strain, this protocol was repeated five to seven times.

### Oviposition

We assessed the oviposition rate of the females exposed to the four humidity treatments over 4 days. We counted the number of eggs laid by each female after 24, 48, 72, and 96 h of exposure. For each humidity treatment and each strain, the oviposition experiment over 4 days was repeated five to ten times, with 15–20 females per replicate.

We also assessed the oviposition rate of females exposed to humidity treatments “constant low”, “constant high”, and “variable 1” during 10 days. Females from the commercial strain were exposed to the three humidity treatments, while females from the mixed strain were exposed to treatment “variable 1” only. One replicate consisted of 20 females and we carried out three replicates per treatment and per strain. On days 6 and 7, females exposed to humidity treatment “variable 1” spent 5 h at low humidity (photoperiod L5:D0) and 19 h at high humidity (photoperiod L11:D8), instead of 8 h at low humidity and 16 h at high humidity, for logistic reasons. The cups from humidity treatment “constant high” were changed after 5 days, to avoid development of fungi inside the cups.

### Survival

The same females used in the oviposition experiment over 10 days were used for the survival experiment: after an exposure of 10 days to humidity treatments “constant low”, “constant high”, and “variable 1”, females were kept under the same conditions during ten additional days. Their survival rate was assessed over these 20 days. Females were supplied every day with ample fresh *T. urticae* (larvae, nymphs and adults) as food. On days 6, 7, 13, 14, and 20, females exposed to humidity treatment “variable 1” spent 5 h at low humidity (photoperiod L5:D0) and 19 h at high humidity (photoperiod L11:D8), instead of 8 h at low humidity and 16 h at high humidity, for logistic reasons. The cups from humidity treatment “constant high” were changed every 5 days, and the cups from humidity treatment “variable 1” were changed after 10 days, to avoid development of fungi inside the cups. To make sure that the potential stress caused by the transfer of the females to new cups did not affect their survival or oviposition rate, we also changed the cups of humidity treatments “constant low” and “variable 1” every 5 days during 20 days, for one replicate. We did not observe an influence of changing cups on the survival or oviposition of the females.

### Statistical analysis

For the egg hatching experiment, we carried out three analyses. In a first model, we studied the effect of the factors humidity treatment (“constant low”, “constant high”, and “variable 1”), strain, and exposure time of females on the hatching rate of *P. persimilis* eggs during 102 h. We also looked at the following interactions, “humidity treatment × exposure time of females” and “humidity treatment × strain”. In a second model, we compared the effects of the humidity treatments “constant low” and “variable 2”, as well as the factors strain and exposure time of females, on the egg hatching rate during the first 78 h (in both treatments, females were exposed to constant low humidity during the first 78 h). We also looked at the interaction “humidity treatment × strain”. We then compared, in a third model, the effects of the two humidity treatments “constant low” and “variable 2”, as well as the factor strain, on the egg hatching rate after 102 h of female exposure (between 78 and 102 h of exposure, humidity increased in treatment “variable 2”). We also looked at the interaction “humidity treatment × strain”. In all three analyses, the response variable was expressed as a proportion (number of eggs hatched/number of eggs tested) for each replicate. For all analyses, we used a generalized linear mixed model (GLMM) with a binomial error distribution and a logit link function. The variables humidity treatment, exposure time of females, and strain were expressed as fixed effects in the models. The replicates which had been performed at the same date were assigned to the same replicate number. The variable replicate was expressed as a random effect in the models (by-replicate random intercept), to take into account the fact that individuals within the same replicate were potentially correlated. Since there was overdispersion in the data for the first two models, we introduced a “per-observation” random effect. For all analyses, we used the model-fitting method of the maximum likelihood (Laplace approximation) and used likelihood-ratio tests to select the most parsimonious models.

For the oviposition experiment, we first analysed the total number of eggs laid per female over 4 days, comparing humidity treatments “constant low”, “constant high”, and “variable 1” for both strains. We also looked at the interaction “humidity treatment × strain”. Data from females which died before the fourth day of the experiment were not used. In total, data from 586 females were analysed. Thereafter, we analysed the total number of eggs laid per female over 10 days, comparing the three humidity treatments (“constant low”, “constant high”, “variable 1”) for the commercial strain, and comparing both strains for humidity treatment “variable 1”. Data from females which died before the tenth day of the experiment were not used. In total, data from 135 females were analysed for the commercial strain, and from 53 females for the mixed strain. For both analyses, we used a generalized linear model (GLM) with a Poisson error distribution and a log link function. We considered a multiplicative dispersion parameter in the variance (quasi-Poisson error distribution), and we found an estimated parameter smaller than 1; therefore, we decided upon a Poisson model with dispersion parameter equal to 1, leading to more conservative conclusions. The variables humidity treatment, strain and replicate were included as fixed effects in the two models. In the analysis of oviposition rate over 10 days, we had only three replicates for each combination of humidity treatment and strain, making a reliable estimate of variance between replicates tenuous (Crawley 2002). Therefore, we specified the variable replicate as a fixed rather than a random effect in the model. Similarly, in the analysis of oviposition rate over 4 days, we included the variable replicate as a fixed effect. We carried out pairwise comparisons of means (Tukey test) to assess differences within strain and humidity treatment levels. For both models, the estimated values for each humidity treatment and strain were obtained by calculating the weighted average of the estimated values of all replicates. The third part of the analysis was about the oviposition experiment over 4 days. For both strains, we calculated for each female the ratio of eggs laid on day 4 divided by the total number of eggs laid over 4 days. We then studied the effects of humidity treatment (“constant low”, “constant high”, “variable 1”, and “variable 2”) and strain on this ratio. We also looked at the interaction “humidity treatment × strain”. Data from females which died before the fourth day of the experiment were not used. In total, data from 728 females were analysed. We used a GLM with a binomial error distribution and a logit link function. The variables humidity treatment, strain and replicate were expressed as fixed effects in the model. The estimated values for each humidity treatment were obtained by calculating the weighted average of the estimated values of all the replicates. We carried out pairwise comparisons of means (Tukey test) to assess differences within humidity treatment levels.

For the survival experiment, we studied the influence of the factors humidity treatment and strain on the survival probability (“time to death”, observed right-censored data) of *P. persimilis* females, during 20 days. The data were right censored because the females that were still alive at day 20 or died from “handling accidents” during the experiment were censored. For each humidity treatment and each strain, all females that were observed during the same 20 days were grouped under the same replicate number. To account for a possible correlation between observations grouped in the same replicate, we used a shared gamma frailty model, with gamma-distributed shared frailties at replicate level (Rondeau et al. 2012). We plotted the estimated survival curves for each humidity treatment using the Kaplan–Meier method (packages survival and survminer in R).

The statistical analysis was performed in R (R version 3. 5. 1).

## Results

### Egg hatching

**Fig. 2:**
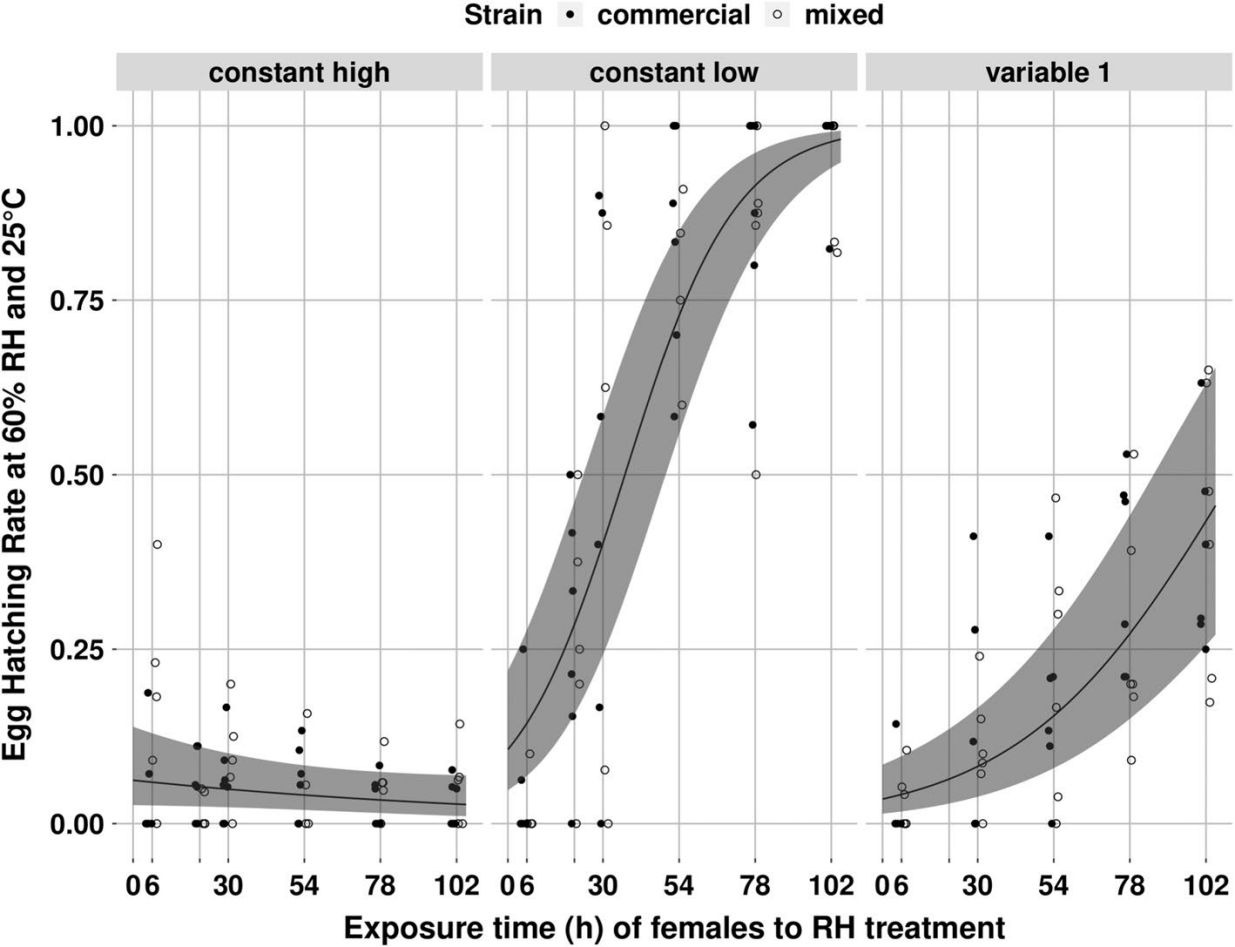
Observed (symbols) and estimated (curves, GLMM) egg hatching rates at 60% RH in relation to the exposure time of *Phytoseiulus persimilis* females to three humidity treatments. One symbol represents one replicate. Full symbols: commercial strain, empty symbols: mixed strain. Symbols are jittered around each exposure time point for more clarity. The shaded areas represent the 95% confidence intervals

**Fig. 3:**
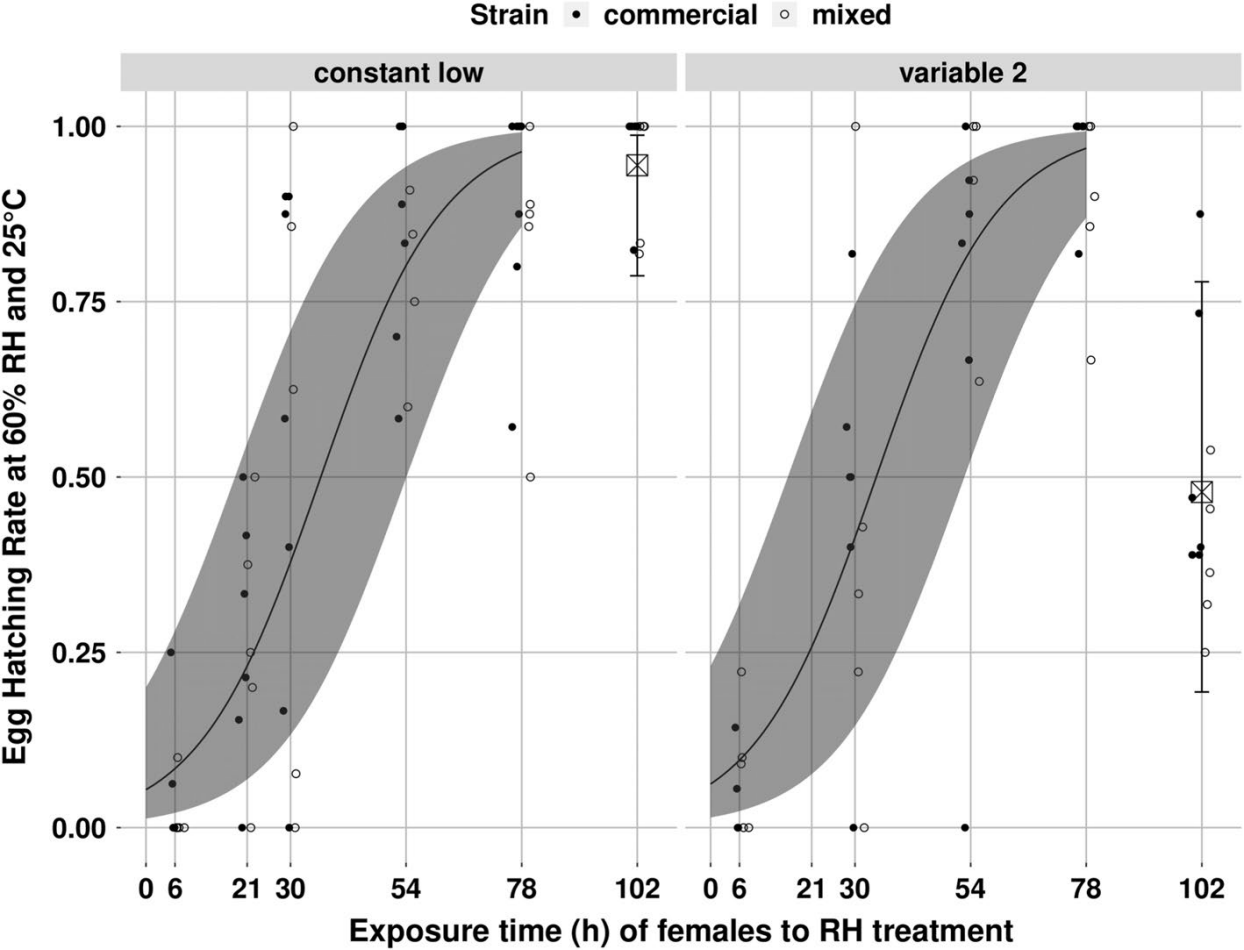
Observed (circular symbols) and estimated (2 curves and 2 square symbols, GLMM) egg hatching rates at 60% RH in relation to the exposure time of *Phytoseiulus persimilis* females to two humidity treatments. One symbol represents one replicate. Full circular symbols: commercial strain, empty circular symbols: mixed strain. Symbols are jittered around each exposure time point for more clarity. The shaded areas represent the 95% confidence intervals

Humidity treatment of *P. persimilis* females significantly affected the hatching rate of their eggs at 60% RH (*χ*^2^ = 7.38; *df* = 2; *P* = 0.02, Fig. 2). This effect became stronger with duration of female exposure, and the interaction between humidity treatment and duration of exposure to the treatment was statistically significant (*χ*^2^ = 75.62; *df* = 2; *P* < 2 × 10^−16^). While the hatching rate of eggs laid by females exposed to constant high humidity remained between 0.03 (− 0.02 to + 0.04) and 0.06 (− 0.03 to + 0.08) (estimated values ± asymmetrical 95% CI) during the whole experiment, hatching rate of eggs laid by females exposed to constant low humidity increased from 0.11 (− 0.06 to + 0.12) after 1 h of exposure, to 0.98 (− 0.04 to + 0.01) after 102 h of exposure. The hatching rate of eggs laid by females exposed to treatment “variable 1” increased to a lesser extent: from 0.04 (− 0.03 to + 0.05) after 1 h of exposure to 0.43 (− 0.18 to + 0.20) after 102 h of exposure. Strain did not significantly affect egg hatching rate (*χ*^2^ = 0.33; *df* = 1; *P* = 0.57) and there was no significant interaction between effects of humidity treatment and strain (*χ*^2^ = 4.89; *df* = 2; *P* = 0.09). For humidity treatment “variable 2”, during the first 78 h of exposure, egg hatching rate was the same as for humidity treatment “constant low” (*χ*^2^ = 0.23; *df* = 1; *P* = 0.63, Fig. 3), and strain had no effect on egg hatching rate (*χ*^2^ = 0.16; *df* = 1; *P* = 0.68). However, after 102 h of female exposure to treatment “variable 2” (24 h after the females had been transferred from low to high humidity), egg hatching rate suddenly decreased. After 102 h of female exposure, hatching rate of eggs laid in treatment “variable 2” (0.48; − 0.29 to + 0.3) was significantly lower (*χ*^2^ = 72.62; *df* = 1; *P* < 2 × 10^−16^) than hatching rate of eggs laid in treatment “constant low” (0.94; − 0.15 to + 0.05) (estimated values ± asymmetrical 95% CI, Fig. 3). Strain did not significantly affect egg hatching rate after 102 h of exposure (*χ*^2^ = 2.45; *df* = 1; *P* = 0.12), and there was no significant interaction between effects of humidity treatment and strain (*χ*^2^ = 0.24; *df* = 1; *P* = 0.62). It is important to notice that, in the egg hatching experiment, we collected around one egg per female after 6, 21, 30, 30, 54, 78, and 102 h of exposure to each humidity treatment. Thus, the proportion of drought-resistant eggs reported here (egg hatching rate at 60% RH) strongly correlates with the percentage of females that laid drought-resistant eggs.

### Oviposition rate

**Fig. 4:**
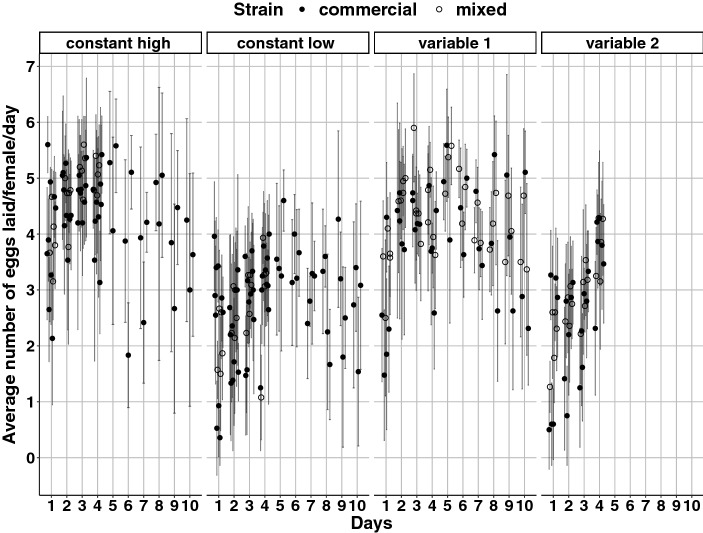
Observed values of the average number of eggs laid by *Phytoseiulus persimilis* females over 10 days and 4 days, when exposed to four humidity treatments, for two strains (full symbols: commercial strain, empty symbols: mixed strain). Each symbol represents one replicate of 15–20 females. The error bars represent ± 1 SD

**Fig. 5:**
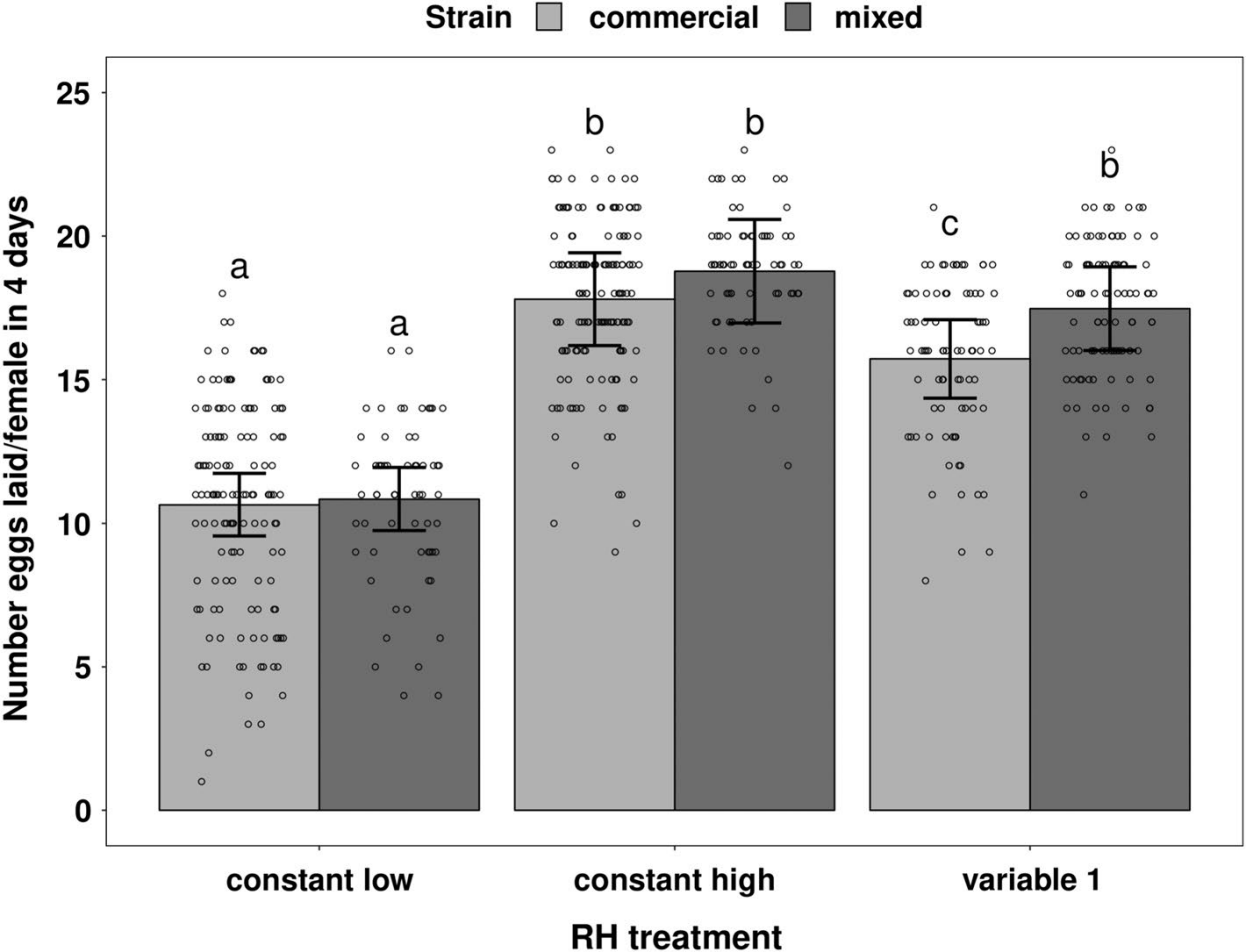
Estimated means (GLM) of the total number of eggs laid by *Phytoseiulus persimilis* females over 4 days, when exposed to three humidity treatments, for two strains (light grey: commercial strain, dark grey: mixed strain). Each dot represents the observed mean for one female. The error bars represent the 95% confidence intervals of the estimated means. Different letters above bars indicate significant differences between treatments and strains (*P* < 0.05)

Humidity treatment significantly affected the oviposition rate over 4 days (*χ*^2^ = 430.28; *df* = 2; *P* < 2 × 10^−16^, Figs. 4 and 5) and the oviposition rate over 10 days (*χ*^2^ = 136.02; *df* = 2; *P* < 2 × 10^−16^, Fig. 4 and Online Resource 1). On average, over 10 days, a female from the commercial strain laid 30.9 ± 2.5 eggs under constant low humidity, 46.2 ± 3.2 eggs under constant high humidity, and 39.5 ± 2.4 eggs under humidity treatment “variable 1” (estimated values ± 95% CI). Strain had a statistically significant effect on the oviposition rate over 4 days (*χ*^2^ = 8.27; *df* = 1; *P* = 0.004), and there was no significant interaction between effects of humidity treatment and strain (*χ*^2^ = 0.07; *df* = 2; *P* = 0.97). On average, over 4 days of exposure to humidity treatment “variable 1”, females from the mixed strain laid significantly more eggs than females from the commercial strain (*P* = 0.046). To investigate further this strain effect on oviposition rate, we compared the oviposition rate of the two strains over 10 days for humidity treatment “variable 1”. The factor strain also had a significant effect on the oviposition rate over 10 days (*χ*^2^ = 7.33; *df* = 1; *P* = 0.007). On average, over 10 days under humidity treatment “variable 1”, a female from the mixed strain laid 3.4 eggs more than a female from the commercial strain.

Finally, humidity treatment had a statistically significant effect on the ratio “number of eggs laid on day 4/number of eggs laid over 4 days” (*χ*^2^ = 47.39; *df* = 3; *P* = 2 × 10^−10^). More specifically, this ratio was significantly different between humidity treatment “variable 2” and the three other treatments (“constant high”, *P* < 0.001; “constant low”, *P* = 0.007; “variable 1”, *P* < 0.001). Females exposed to humidity treatment “variable 2” laid a significantly higher number of eggs during the last 24 h compared to the first 78 h, whereas for the three other treatments the number of eggs produced stayed relatively constant over 4 days. On average, 34% (− 4% to + 5%) of the eggs laid by a female exposed to humidity treatment “variable 2” were laid during the last 24 h, while between 25% and 29% of the eggs laid by a female exposed to humidity treatments “constant low” (29%; − 5% to + 4%), “constant high” (26%; − 4% to + 3%), or “variable 1” (25%; − 4% to + 3%) were laid during the last 24 h (estimated values ± asymmetrical 95% CI). Strain had no effect on this ratio (*χ*^2^ = 3.05; *df* = 1; *P* = 0.08), and there was no significant interaction between effects of humidity treatment and strain (*χ*^2^ = 7,8; *df* = 3; *P* = 0.05). Overall, the oviposition rate experiment showed that *P. persimilis* females lay fewer eggs when exposed to constant low humidity than when exposed to constant high or variable humidity.

### Adult survival

**Fig. 6:**
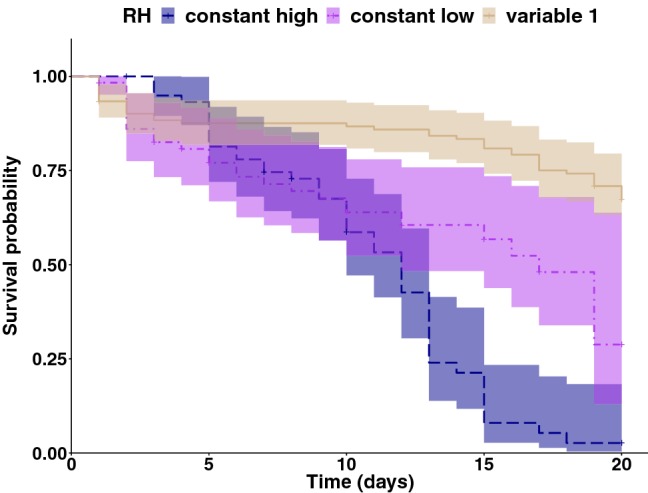
Survival probabilities for *Phytoseiulus persimilis* females exposed to three humidity treatments at 25 °C during 20 days. Three replicates per treatment and 20 females per replicate (Kaplan–Meier method, with 95% CI)

The humidity treatment had a statistically significant effect on the survival of *P. persimilis* females over 20 days (*χ*^2^ = 31.22; *df* = 2; *P* = 2 × 10^−7^, Fig. 6). Females exposed to humidity treatment “variable 1” survived significantly longer than females exposed to constant low and constant high humidity (*P* = 1 × 10^−4^ for “constant low”; *P* = 2 × 10^−8^ for “constant high”). Around 70% of *P. persimilis* females were still alive after 20 days under humidity treatment “variable 1”, whereas only less than 35% and 25% of the females survived after 20 days under constant low and constant high humidity, respectively. Females exposed to constant low humidity survived a bit longer than the ones exposed to constant high humidity; however, this difference was not statistically significant (*P* = 0.07). Strain did not affect survival of the females under humidity treatment “variable 1” (*P* = 0.32). Overall, the survival experiment showed that the lifespan of *P. persimilis* females is shorter under constant low and constant high humidity, compared to variable humidity conditions.

## Discussion

Our data show that the relative humidity experienced by *P. persimilis* females has a strong effect on drought resistance of their eggs. The size of this effect depends on the duration of female exposure. When females are exposed to constant low relative humidity (65% RH at 25 °C) during 102 h, almost all females lay drought-resistant eggs. However, when females are exposed to constant high relative humidity (95% RH at 25 °C) during 102 h, only 3–6% lay drought-resistant eggs. These results demonstrate a strong phenotypic plasticity in *P. persimilis* females with regard to relative humidity: they are capable of sensing unfavourable humidity conditions, and prenatally prepare their offspring to cope with drought. This mechanism for transgenerational phenotypic plasticity, or maternal effect, is defined as the ability of a female to alter its offspring’s phenotype, allowing it to survive in a specific environment (Bernardo 1996; Mousseau and Fox 1998; Fox et al. 1999; Freinschlag and Schausberger 2016). In phytoseiid mites, maternal strategies promoting offspring survival have been associated with food deprivation, risk of intraguild predation, and risk of sibling cannibalism (Toyoshima and Amano 1998; Schausberger and Hoffmann 2008; Walzer and Schausberger 2011, 2015; Seiter and Schausberger 2015). To our knowledge, only one published study has reported a maternal effect associated with relative humidity in mites: in larvae of the American dog tick *Dermacentor variabilis* (Say) (Acari: Ixodidae), the ability to absorb water vapor from the air is under maternal control (Yoder et al. 2006). This maternal effect has the adaptive significance of enabling larvae to maintain adequate levels of body water, by preventing dehydration and over-hydration.

In our study, the maternal effect observed in *P. persimilis* females takes the form of a discrete or switched response. When *P. persimilis* females exposed to low humidity for 78 h were transferred back to high humidity for 24 h, the percentage of females laying drought-resistant eggs significantly decreased in the last 24 h, from 90% to 48%. This sudden drop suggests that the production of drought-resistant eggs in *P. persimilis* females is the result of a maternal investment that can quickly be ‘switched’ on and off. From there, two questions arise: how does the production of drought-resistant eggs affect *P. persimilis* females, more particularly their oviposition and survival rates? And how do *P. persimilis* females find the most ‘adaptive’ strategy to ensure the survival of their eggs as well as their own survival in dry conditions?

Our data show that the oviposition rate of *P. persimilis* females depends on relative humidity conditions in their environment. Most females exposed to low humidity for 78 h significantly increased their oviposition rate after being transferred to high humidity for 24 h. Moreover, females exposed to constant low humidity had the lowest oviposition rate. We propose two non-exclusive explanations for this. First, this may relate to the costs of laying drought-resistant eggs. Females exposed to constant low humidity laid the highest proportion of drought-resistant eggs. Drought resistance in arthropods can be achieved through three main mechanisms: an increase in initial body water content, a decreased water loss rate, or a higher drought tolerance, i.e. the capacity to tolerate the loss of a higher percentage of water prior to death (Gefen et al. 2006; Bazinet et al. 2010). Although the mechanisms making *P. persimilis* eggs drought resistant remain to be elucidated, the production of such eggs may represent a higher energetic cost for the females, resulting in a trade-off between quality and quantity of offspring (Fox et al. 1999; Moczek 2010). Second, the low oviposition rate under constant low humidity may relate to investment of the females in their own survival. *Phytoseiulus persimilis* females can carry only one mature egg at a time, since it requires a considerable amount of energy and resources: a *P. persimilis* egg weighs more than 20% of the female body weight (Sabelis 1981). Therefore, the decrease we observed in oviposition rate under low humidity may also be the result of a reallocation of the resources to somatic maintenance, instead of reproduction (Montserrat et al. 2007). For example, when prey density varies, *P. persimilis* females adjust the number of eggs deposited in a prey patch to their own nutritional needs, enhancing adult survival and reproduction, and to the needs of their progeny, enhancing immature survival and development (Vanas et al. 2006). Under humidity treatment “variable 1” (exposure to low humidity for 1/3 of the time), around 43% of the females laid drought-resistant eggs after 102 h of exposure. Interestingly, oviposition rate under treatment “variable 1” was not significantly different (mixed strain) or slightly significantly lower (commercial strain) compared to oviposition rate of females exposed to constant high humidity, which did not lay drought-resistant eggs. These results suggest that fecundity is not proportionally affected by the exposure time to low humidity. Moreover, females exposed to humidity treatment “variable 1” probably did not suffer from dehydration and did not need to reallocate resources to their own survival. Their oviposition rate was, therefore, only affected by the production of a limited proportion of drought-resistant eggs and modified to a lesser extent. On the contrary, females exposed to humidity treatment “constant low” had to deal with the combination of a severe drought stress and the production of a high proportion of drought-resistant eggs, resulting in a significant decrease in oviposition rate.

The only difference we observed between the two strains tested was in the oviposition rate over 10 days under humidity treatment “variable 1”: the mixed strain laid on average 3.4 eggs more than the commercial strain. However, the biological significance of these 3.4 eggs over 10 days in terms of impact on population dynamics is likely limited. Despite various differences between these two strains (rearing conditions, origin), they had a similar response to the relative humidity treatments tested in this study. These results indicate that there was probably little variation for the production of drought-resistant eggs between these strains and that there might be little room for improvement of the adaptive maternal strategies described previously.

We also showed that *P. persimilis* female lifespan depends on relative humidity conditions in their environment. We recorded a shorter lifespan for *P. persimilis* females exposed to constant low humidity. More surprisingly, females exposed to constant high humidity had the shortest lifespan. A previous study showed that when both *T. urticae* and *P. persimilis* were exposed to 100% RH, their activity diminished gradually with time, and more than 90% of the mites ceased activity after 4 h (Mori and Chant 1966). For *T. urticae*, water loss by evaporation through the cuticle is necessary because it allows the ingestion of large quantities of plant liquids and the concentration of the nutrients in the mite’s body (Boudreaux 1958). Extremely high relative humidity (95–100%), therefore, has a negative effect on feeding of *T. urticae*, by preventing loss of moisture from the body by evaporation. In our experiment, *P. persimilis* females exposed to 95% RH did not cease activity, since they had the highest oviposition rate of all three treatments. However, they might have decreased their predation rate, due to a water retention problem, similar to that of *T. urticae*. This water retention problem, combined with other unknown physiological effects, probably contributed to shorten the lifespan of *P. persimilis* females under extreme high humidity.

After 102 h of exposure to humidity treatment “variable 1”, around 43% of the females laid drought-resistant eggs. This observation is somewhat surprising, considering the humidity conditions needed for a non-drought-resistant *P. persimilis* egg to successfully hatch. More than 50% of *P. persimilis* eggs laid under high humidity conditions successfully hatch under low humidity (60% RH at 25 °C) if these eggs are exposed to high humidity (75% RH at 25 °C) for at least 7 h during their development (Le Hesran et al. 2019). Moreover, under successive cycles of 12 h at low and 12 h at high humidity, more than 75% of *P. persimilis* eggs hatch (Le Hesran et al. 2019). Therefore, in our experiment, *P. persimilis* females exposed to humidity treatment “variable 1” should not have perceived these conditions as particularly unfavourable for their eggs. Still, 43% of them switched and started laying drought-resistant eggs after 102 h of exposure. Possibly, a small risk of mortality in *P. persimilis* females’ offspring is already enough to change the type of eggs they lay. Moreover, environmental variations are highly stochastic. For a female experiencing some unpredictability in the environment, it may be more adaptive to prepare her offspring for the worst conditions, therefore laying drought-resistant eggs even when relative humidity conditions are not so unfavourable. Theory shows that a ‘bet-hedging’ strategy is successful when facing unpredictability. This strategy uses the idea of “not putting all your eggs in the same basket” and results in limited variation in long-term offspring success (Rossiter et al. 1993). Under variable humidity conditions, *P. persimilis* females may use this strategy and produce drought-resistant and non-drought-resistant eggs alternately to maximize their contribution to the next generation. We can, therefore, hypothesize that the 43% females who laid drought-resistant eggs after 102 h under treatment “variable 1” may have laid a non-drought-resistant egg subsequently. This strategy of alternating between drought-resistant and non-drought-resistant eggs under variable humidity may explain why around half of the eggs hatched under these conditions. Under constant low humidity conditions, where *P. persimilis* eggs are expected to die, a different strategy is adopted by *P. persimilis* females, which seems to be the most adaptive for both offspring and maternal fitness: laying drought-resistant eggs only, while maintaining the health of the mother through a decrease in oviposition rate. When relative humidity rises again, a high level of plasticity in the production of drought-resistant eggs is adaptive for *P. persimilis* females. Under constant high humidity, most *P. persimilis* eggs are expected to hatch successfully, and *P. persimilis* females have no need to lay drought-resistant eggs.

Although these maternal strategies seem to be beneficial for *P. persimilis* females and their offspring, we observed an interesting phenomenon: not all females started laying drought-resistant eggs, or switched, at the same time. The observation that females from the same strain, same age, and exposed to the same conditions responded differentially is intriguing and raises two questions: first, what is the humidity threshold separating the production of non-drought-resistant eggs from the production of drought-resistant eggs in *P. persimilis* females? Second, is this threshold the same for all individuals? Between 6 and 21 h of exposure to constant low humidity, the percentage of females laying drought-resistant eggs increased from 14.4% to 28.5%. Under humidity treatment “variable 1”, however, where females spent 8 h per day at low humidity, it took 80 h of exposure to reach the same proportion of females laying drought-resistant eggs. We therefore hypothesize that, in terms of threshold, *P. persimilis* females need a minimum of 6–21 h of constant exposure to low humidity (65% RH at 25 °C) to start laying drought-resistant eggs and that an interruption of this exposure will delay the response of the females.

The second question was on the variation of the humidity threshold separating the production of non-drought-resistant eggs from the production of drought-resistant eggs between females. Why did some females start laying drought-resistant eggs after 24 h of exposure to low humidity, whereas others only did so after 78 h of exposure? There could be two reasons for this. First, each female may respond to variation in humidity according to her own physiological limits, and her own degree of plasticity. However, much of the behavioural variation within populations cannot be attributed to within-individual plasticity and physiology alone (Dingemanse et al. 2010). A second reason for this behavioural variation could be that each *P. persimilis* female has her own life-experience and personality, influencing her behaviour (Gosling 2001; Dall et al. 2004; Biro and Stamps 2008; McNamara et al. 2008; Wolf 2009). The concept of personality has been used for *P. persimilis*. Early social isolation, for example, has proven to impair development, mate choice and grouping behaviour of *P. persimilis* and, therefore, to be an important determinant in shaping *P. persimilis* individual personality (Schausberger et al. 2017).

In conclusion, our study provides new insight into the effects of relative humidity on the predatory mite *P. persimilis*. We show that a maternal effect of *P. persimilis* females determines egg survival when females are exposed to constant low and variable humidity conditions: they produce drought-resistant eggs. The production of drought-resistant *P. persimilis* eggs is a phenotypically highly plastic trait, and the mechanisms making them drought resistant still remain to be elucidated.

## Electronic supplementary material

Below is the link to the electronic supplementary material.

Supplementary material 1 (PDF 114 kb)
