# Supplementary material for: Maternal effect determines drought resistance of eggs in the predatory mite Phytoseiulus persimilis
Source: Oecologia. 2019 Nov 26;192(1):29–41. doi: 10.1007/s00442-019-04556-0 (PMC6974492; doi:10.1007/s00442-019-04556-0)
Supplement: Supplementary file 1 — Supplementary material 1 (PDF 114 kb) [file 442_2019_4556_MOESM1_ESM.pdf]

Maternal effect determines drought resistance of eggs in the predatory mite *Phytoseiulus persimilis*

Sophie Le Hesran[1,2,*], Thomas Groot[1], Markus Knapp[1], Tibor Bukovinszky[1], Jovano Erris Nugroho[1], Giuditta Beretta[1], Marcel Dicke[2]

Corresponding author: sophielehesran@gmail.com (S. Le Hesran)

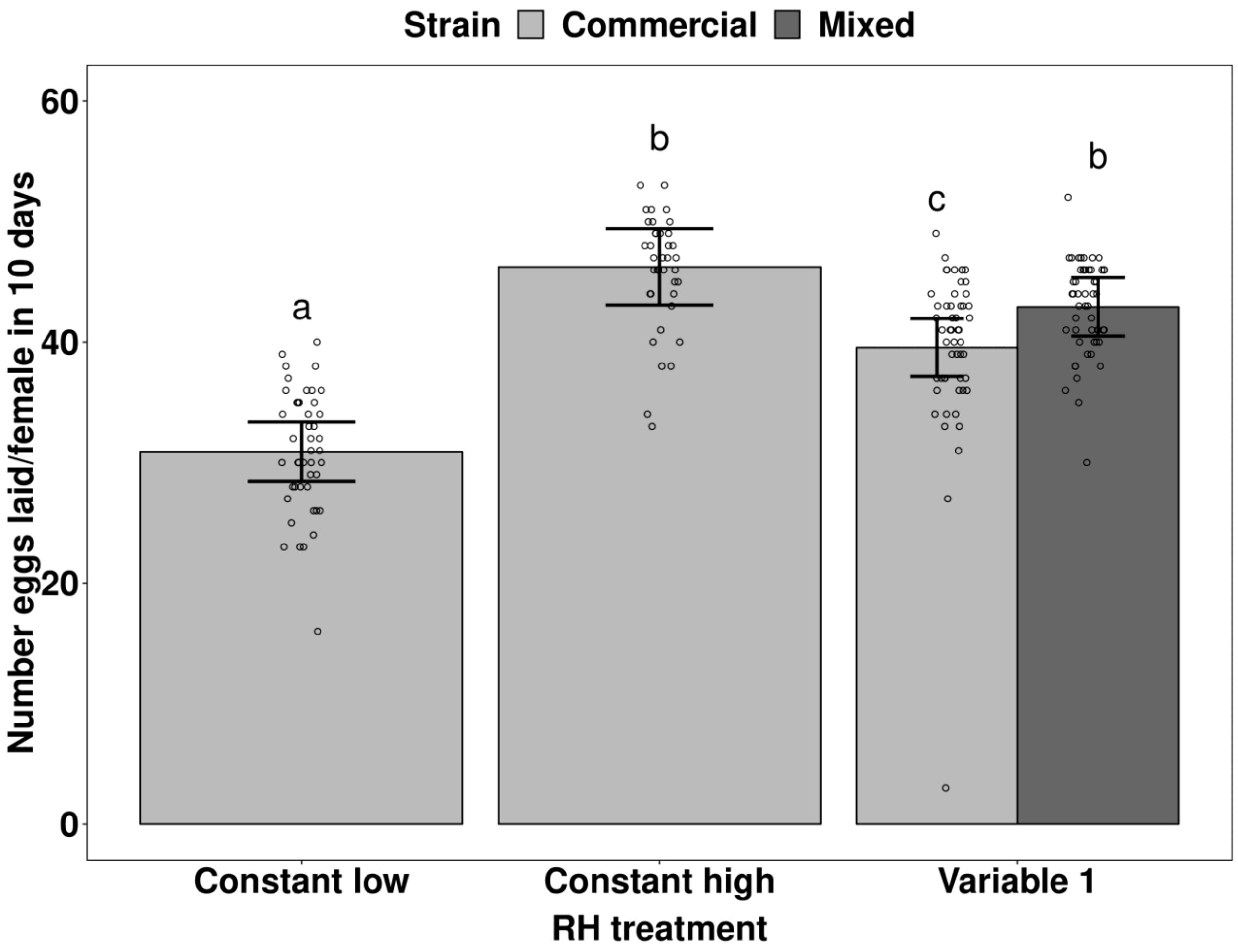

Online Resource 1: Estimated means (GLM) of the total number of eggs laid by *P. persimilis* females over ten days, when exposed to three humidity treatments, for two strains (light grey: commercial strain, dark grey: mixed strain). Each dot represents the observed mean for one female. The error bars represent the 95% confidence intervals of the estimated means. Different letters above bars indicate significant differences between treatments and strains ($P < 0.05$).
